# CD137 Expression Is Induced by Epstein-Barr Virus Infection through LMP1 in T or NK Cells and Mediates Survival Promoting Signals

**DOI:** 10.1371/journal.pone.0112564

**Published:** 2014-11-19

**Authors:** Mayumi Yoshimori, Ken-Ichi Imadome, Honami Komatsu, Ludan Wang, Yasunori Saitoh, Shoji Yamaoka, Tetsuya Fukuda, Morito Kurata, Takatoshi Koyama, Norio Shimizu, Shigeyoshi Fujiwara, Osamu Miura, Ayako Arai

**Affiliations:** 1 Department of Hematology, Graduate School of Medical and Dental Sciences, Tokyo Medical and Dental University, Tokyo, Japan; 2 Department of Laboratory Molecular Genetics of Hematology, Graduate School of Health Care Sciences, Tokyo Medical and Dental University, Tokyo, Japan; 3 Department of Infectious Diseases, National Research Institute for Child Health and Development, Tokyo, Japan; 4 Department of Molecular Virology, Graduate School of Medical and Dental Sciences, Tokyo Medical and Dental University, Tokyo, Japan; 5 Department of Comprehensive Pathology, Graduate School of Medical and Dental Sciences, Tokyo Medical and Dental University, Tokyo, Japan; 6 Department of Virology, Division of Medical Science, Medical Research Institute, Tokyo Medical and Dental University, Tokyo, Japan; The University of North Carolina at Chapel Hill, United States of America

## Abstract

To clarify the mechanism for development of Epstein-Barr virus (EBV)-positive T- or NK-cell neoplasms, we focused on the costimulatory receptor CD137. We detected high expression of *CD137* gene and its protein on EBV-positive T- or NK-cell lines as compared with EBV-negative cell lines. EBV-positive cells from EBV-positive T- or NK-cell lymphoproliferative disorders (EBV-T/NK-LPDs) patients also had significantly higher *CD137* gene expression than control cells from healthy donors. In the presence of IL-2, whose concentration in the serum of EBV-T/NK-LPDs was higher than that of healthy donors, CD137 protein expression was upregulated in the patients' cells whereas not in control cells from healthy donors. *In vitro* EBV infection of MOLT4 cells resulted in induction of endogenous CD137 expression. Transient expression of *LMP1*, which was enhanced by IL-2 in EBV-T/NK-LPDs cells, induced endogenous *CD137* gene expression in T and NK-cell lines. In order to examine *in vivo* CD137 expression, we used EBV-T/NK-LPDs xenograft models generated by intravenous injection of patients' cells. We identified EBV-positive and CD8-positive T cells, as well as CD137 ligand-positive cells, in their tissue lesions. In addition, we detected CD137 expression on the EBV infected cells from the lesions of the models by immune-fluorescent staining. Finally, CD137 stimulation suppressed etoposide-induced cell death not only in the EBV-positive T- or NK-cell lines, but also in the patients' cells. These results indicate that upregulation of CD137 expression through LMP1 by EBV promotes cell survival in T or NK cells leading to development of EBV-positive T/NK-cell neoplasms.

## Introduction

Epstein-Barr virus (EBV) infection can be found in lymphoid malignancies not only of B-cell lineage, but also of T- or NK-cell lineages. These EBV-positive T or NK-cell neoplasms, such as extranodal NK/T-cell lymphoma nasal type (ENKL), aggressive NK-cell leukemia (ANKL), and EBV-positive T- or NK- cell lymphoproliferative diseases (EBV-T/NK-LPDs), are relatively rare but lethal disorders classified as peripheral T/NK-cell lymphomas according to the WHO classification of tumors of hematopoietic and lymphoid malignancies. ENKL is a rapidly progressive lymphoma characterized by extranodal lesions with vascular damage and severe necrosis accompanied by infiltration of neoplastic NK or cytotoxic T cells [1]. ANKL is a markedly aggressive leukemia with neoplastic proliferation of NK cells [2]. EBV-T/NK-LPDs is a fatal disorder presenting sustained infectious mononucleosis-like symptoms, hypersensitivity to mosquito bites, or hydroa vacciniforme-like eruption accompanied by clonal proliferation of EBV-infected cells [3], [4]. Because most reported cases were children or young adults, and were mainly of the T-cell-infected type, the disorders were designated “EBV-positive T-cell lymphoproliferative diseases of childhood” in the WHO classification, although adult and NK-cell types have been reported [4]–[6]. The common clinical properties of EBV-T/NK-neoplasms are the presence of severe inflammation, resistance to chemotherapy, and a marked geographic bias for East Asia and Latin America, suggesting a genetic context for disease development [4]. Since these EBV-T/NK-neoplasms overlap [4], common mechanisms are thought to exist in the background and contribute to disease development.

It is well known that EBV infects B cells and makes the infected cells immortal resulting in B-cell lymphomas. Similarly it is suspected that EBV may also cause T- or NK-cell neoplasms. However, why and how EBV latently infects T or NK cells, whether or not EBV directly causes these malignancies, and the mechanism of action responsible for the disease development remain to be clarified. Although new chemotherapy and stem cell transplantation have achieved good results for EBV-T/NK neoplasms recently [7]–[9], prognosis of the diseases is still poor. The mechanisms for development of the disease need to be determined to establish an optimal treatment.

To clarify the molecular mechanism underlying the development of EBV-T/NK-neoplasms, we focused on the costimulatory receptor CD137. CD137, also known as 4-1BB, is a member of the tumor necrosis factor (TNF) receptor superfamily, and expressed on the surface of activated T and NK cells [10]. In association with TCR stimulation, it plays a pivotal role in proliferation, survival, and differentiation of these cells as a costimulatory molecule [11]. Recently, it was reported that CD137 is expressed on tumor cells from adult T-cell leukemia/lymphoma (ATLL) and from T-cell lymphomas [12], [13]. Here we found CD137 expression on EBV-positive cells in EBV-T/NK-neoplasms and investigated its role for the lymphomagenesis using established cell lines as well as cells from EBV-T/NK-LPDs patients.

## Results

### CD137 expression in EBV-T/NK-cell lines

**Figure 1:**
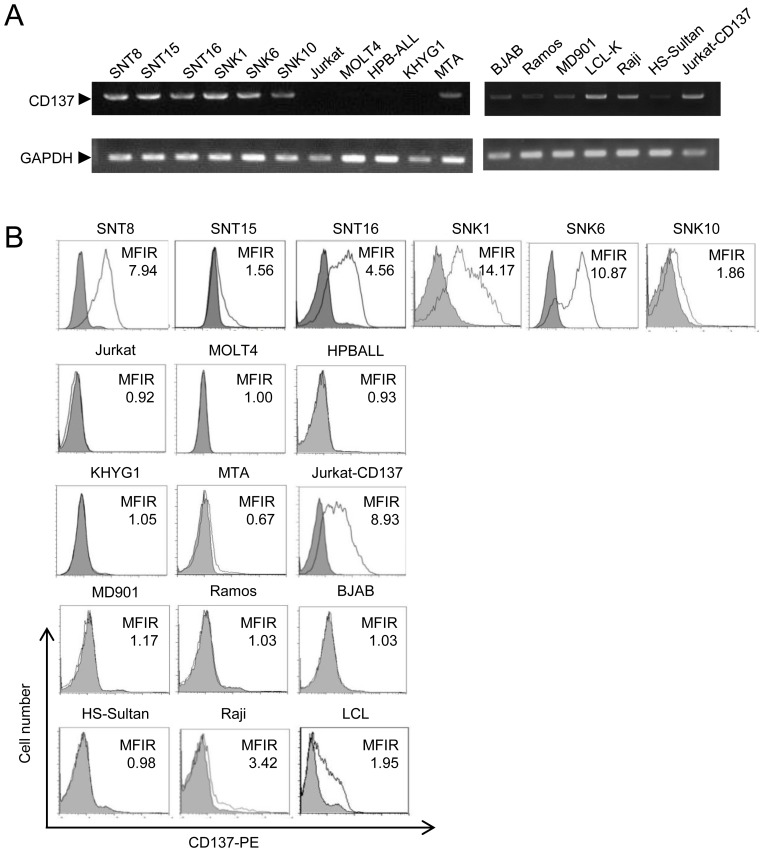
CD137 expression in Epstein-Barr virus (EBV)-positive T- or NK-cell lines. (A) Transcripts of *CD137* (the upper panel) and *GAPDH* (the lower panel) in EBV- positive T- or NK-cell lines were examined by RT-PCR. EBV negative T-, NK, B-cell lines, and EBV-positive B-cell lines were also obtained for the examination. (B) Surface expression of CD137 was examined by flow cytometry using an antibody to CD137 (open histogram) or isotype-matched control immunoglobulin (gray, shaded histogram). The mean fluorescent intensity of CD137 was normalized by that of isotype-matched control and expressed as mean fluorescence intensity rate (MFIR). Each experiment was independently performed more than 3 times and their average data are presented.

Six EBV-positive T- and NK-cell lines, SNT8, SNT15, SNT16, SNK1, SNK6, and SNK10 had been established from primary lesions of ENKL patients (SNT8 and SNK6) and PB of EBV-T/NK-LPDs patients (SNT15, SNT16, SNK1, and SNK10) [14]. We investigated *CD137* mRNA expression in the cell lines by RT-PCR. *CD137* mRNA was expressed in all of them, whereas EBV-negative T-cell lines (Jurkat, MOLT4, and HPB-ALL) and NK-cell line (KHYG1) were negative for the expression (Figure 1A). The mRNA was detected but weak in an EBV-negative NK-cell line, MTA, and in EBV-negative B-cell lines, BJAB, Ramos, and MD901. We also investigated 3 EBV-positive B cell lines, Raji, a lymphoblastoid cell line (LCL), and HS-sultun. The expression was detected in Raji. The expression was weak in LCL, and negative in HS-Sultan. We next investigated CD137 protein expression on the cell surface. Figure 1B shows that CD137 protein was expressed on the cell surface of all EBV-positive T- or NK-cells. In contrast, EBV-negative T-, NK-, and B-cell lines were negative for CD137 expression. On the basis of these results, we concluded that CD137 expression was induced at the mRNA and protein levels in EBV-T/NK cell lines. The expression was detected in 2 of 3 examined EBV-positive B cell lines, Raji and LCL, whereas negative in HS-Sultan. The expression in EBV-positive B cells was insignificant in comparison with EBV-positive T or NK cells. We were unable to detect CD137L expression on the surface these EBV-positive T- or NK-cells lines. The expression was negative on them (Figure S1).

### EBV induces CD137 expression in T and NK cells

**Figure 2:**
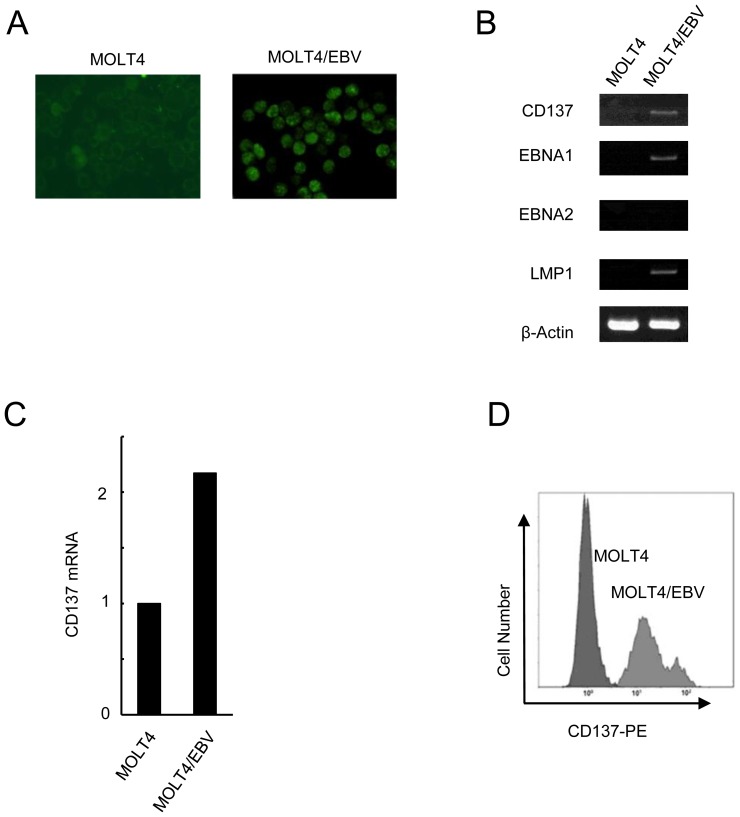
EBV induces CD137 expression in T cells. *In vitro* EBV infection assay performed in MOLT4 cells. (A) EBNA1 protein expression was examined by immune fluorescence staining, 48 hours after the infection, the time when CD137 expression was examined. (B) Expression of the *CD137* gene was examined by RT-PCR. The infection was confirmed by detecting mRNAs of the viral proteins, EBNA1 and LMP1. (C) Transcripts of *CD137* and *GAPDH* were quantified by real time RT-PCR. Relative copy number was obtained by normalizing the *CD137* transcripts to those of those of *GAPDH*. (D) Surface expression of CD137 of MOLT4 cells and EBV-infected MOLT4 cells was examined by flow cytometry.

To clarify whether EBV could directly induce CD137 expression, we performed *in vitro* EBV infection of an EBV-negative cell line MOLT4. EBV DNA copy number of EBV-infected MOTL4 cells was 8.8×10^5^ copies/µgDNA. EBV infection was verified by the presence of EBV nuclear antigen (EBNA) 1 protein expression (Figure 2A). Most cells were positive for EBNA1. The infection was also confirmed by the presence of the viral mRNA, *LMP1* and *EBNA1*, and the absence of *EBNA2* by RT-PCR (Figure 2B). This expression pattern was classified as latency type 2. *CD137* mRNA was also expressed in EBV-infected MOTL4 cells (Figure 2B and 2C). In addition, Figure 2D showed that CD137 protein expression was detected on EBV-infected MOLT4 cells. We therefore concluded that EBV infection induced mRNA and surface protein expression of CD137 in MOLT4 cells.

### CD137 expression in cells from EBV-T/NK-LPDs patients

The above results were validated using EBV-T/NK cells derived from patients. In EBV-T/NK-LPDs, EBV infection could be detected in a particular fraction of PBMCs and isolated at high purity using antibody-conjugated magnetic beads as described in “Materials and Methods”. Seventeen patients (aged 8–72 years; 7 males, 10 females; 10 T- and 7 NK-cell types; CD4 type n = 4, CD8 type n = 5, γδ type n = 1, and CD56 type n = 7) were diagnosed with EBV-T/NK-LPDs according to the criteria as described in “Materials and Methods”. We determined the EBV-positive fraction of the lymphocytes in the PB at the diagnosis. The phenotype of the infected cells and EBV DNA load of them were presented in Table 1. EBV DNA was negative or relatively low in CD19-positive cell which EBV can infect (Table 1).

**Table 1:** Clinical information of the patients' samples subjected to the assay.

Case	Gender	Age	Infected cell	Clinical findings	EBV-DNA (copies/µgDNA) of PB (whole blood)	EBV-DNA (copies/µgDNA) of the EBV-infected cells fraction in PB	EBV-DNA (copies/µgDNA) of CD19-positive cells fraction in PB
CD4-1	M	45	CD4	sCAEBV	3.1×10^2^	4.4×10^4^ (CD4)	4.4×10^2^
CD4-2	F	25	CD4	HMB	7.0×10^4^	2.2×10^5^ (CD4)	N.D.
CD4-3	F	62	CD4	sCAEBV	3.2×10^4^	4.6×10^5^ (CD4)	N.D.
CD4-4	F	72	CD4	sCAEBV	9.4×10^4^	6.4×10^5^ (CD4)	N.D.
CD8-1	F	38	CD8	sCAEBV	1.4×10^5^	3.9×10^5^ (CD8)	N.D.
CD8-2	F	21	CD8	sCAEBV	1.9×10^3^	4.2×10^4^ (CD8)	N.D.
CD8-3	F	64	CD8	sCAEBV	2.6×10^5^	1.2×10^6^ (CD8)	4.6×10^5^
CD8-4	M	28	CD8	sCAEBV	1.9×10^3^	4.1×10^5^ (CD8)	2.0×10^4^
CD8-5	M	13	CD8	sCAEBV	2.1×10^3^	6.4×10^4^ (CD8)	N.D.
γδ	M	9	γδ	HV	8.0×10^3^	2.6×10^4^ (γδ)	N.D.
CD56-1	F	18	CD56	sCAEBV	2.5×10^2^	5.0×10^4^ (CD56)	N.D.
CD56-2	F	13	CD56	HMB	5.2×10^4^	1.6×10^6^ (CD56)	7.5×10^4^
CD56-3	F	23	CD56	sCAEBV	1.0×10^4^	1.1×10^5^ (CD56)	N.D.
CD56-4	F	48	CD56	sCAEBV	8.6×10^4^	1.6×10^5^ (CD56)	N.D.
CD56-5	M	9	CD56	sCAEBV	1.1×10^4^	5.2×10^5^ (CD56)	N.D.
CD56-6	M	8	CD56	sCAEBV	5.1×10^2^	3.5×10^4^ (CD56)	N.D.
CD56-7	M	24	CD56	sCAEBV	2.3×10^3^	2.1×10^4^ (CD56)	N.D.

M: Male, F: Female.

EBV: Epstein-Barr virus, PB: peripheral blood.

sCAEBV: systemic chronic active Epstein-Barr virus infection, HMB: hypersensitivity to mosquito bites (HMB), HV: hydroa vacciniforme-like eruption.

*The clonality was detected by Southern blotting for EBV terminal repeat.

**Figure 3:**
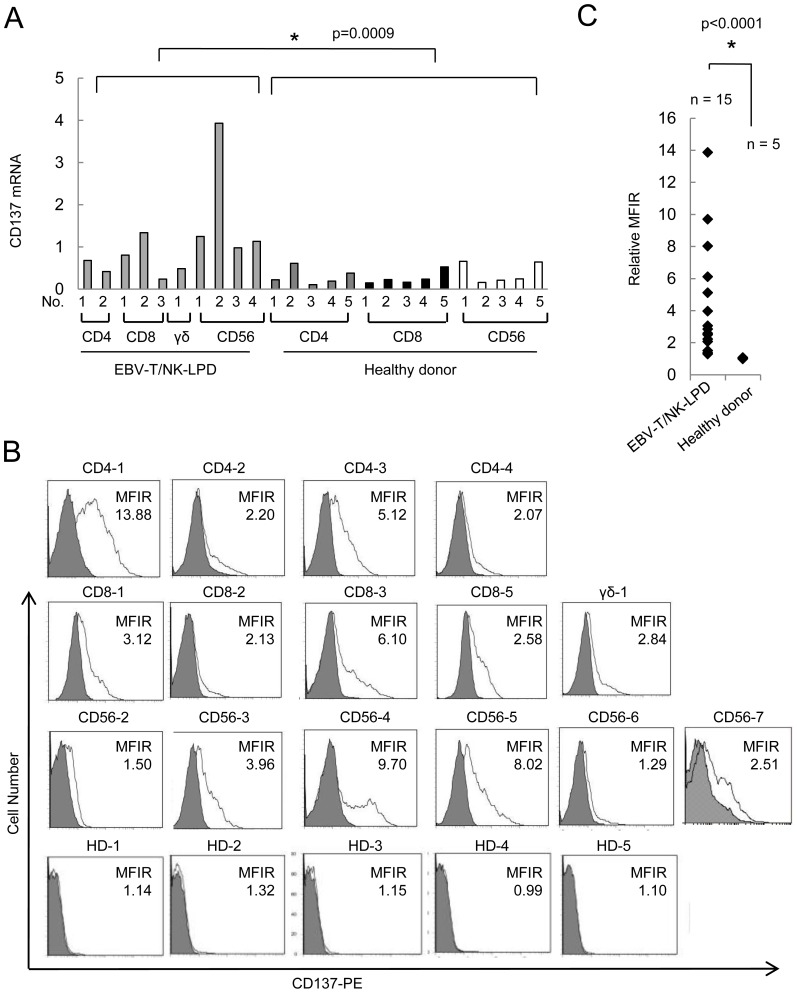
CD137 expression in EBV-positive T or NK cells of patients with EBV-T/NK-lymphoproliferative disorders (EBV-T/NK-LPDs). (A) Transcripts of *CD137* and *GAPDH* of freshly isolated EBV-positive cell fractions from 9 EBV-T/NK-LPDs patients, or cells of the same fractions from healthy donors were quantified by real-time RT-PCR. Relative copy number was obtained by normalizing the *CD137* transcripts to those of *GAPDH*. The relative copy number of the EBV-T/NK-LPDs patients' cells and healthy donor cells were compared. (B) CD137 protein expression in peripheral blood mononuclear cells (PBMCs) from 15 EBV-T/NK-LPDs patients or 5 healthy donors. PBMCs were cultured with IL-2 for 3 days and examined by flow cytometry. The mean fluorescent intensity of CD137 was normalized by that of isotype-matched control and expressed as MFIR (mean fluorescence intensity rate). (C) A bar graph for the relative MFIRs. Each point represents the MFIR of each sample.

To examine CD137 expression in the EBV-positive fraction, the fractions were isolated by the magnetic beads and obtained for *CD137* mRNA detection in 10 patients. Figure 3A shows the *CD137* mRNA levels in the freshly isolated cells of EBV-positive cell fraction in PBMCs of each patient. *CD137* mRNA levels in CD4-, CD8-, and CD56-positive cell fractions of 5 healthy donors' PBMCs were also demonstrated. The mRNA levels in the patients' cells were significantly higher than those in the cells of healthy donors. Next we examined the expression of CD137 protein by flow cytometry. It showed low expression in freshly isolated PBMCs from both patients and 5 healthy donors (data not shown). However, after culture with IL-2 for 3 days, the expression was increased on the surface of PBMCs from 15 patients but still low on the cells isolated from 5 healthy donors (Figure 3B). The average of CD137 protein levels of EBV-T/NK-LPDs patients was significantly higher than that of healthy donors (Figure 3C). Two-color flow cytometry using antibodies to CD137 and to surface proteins expressed on EBV-positive cells could be performed in 7 patients, and a double-staining pattern was observed in them, whereas fractions from a healthy donor barely expressed the CD137 protein. (Figure S2).

### EBV LMP1 induces CD137 expression in T and NK cells through LMP1 induced by IL-2

**Figure 4:**
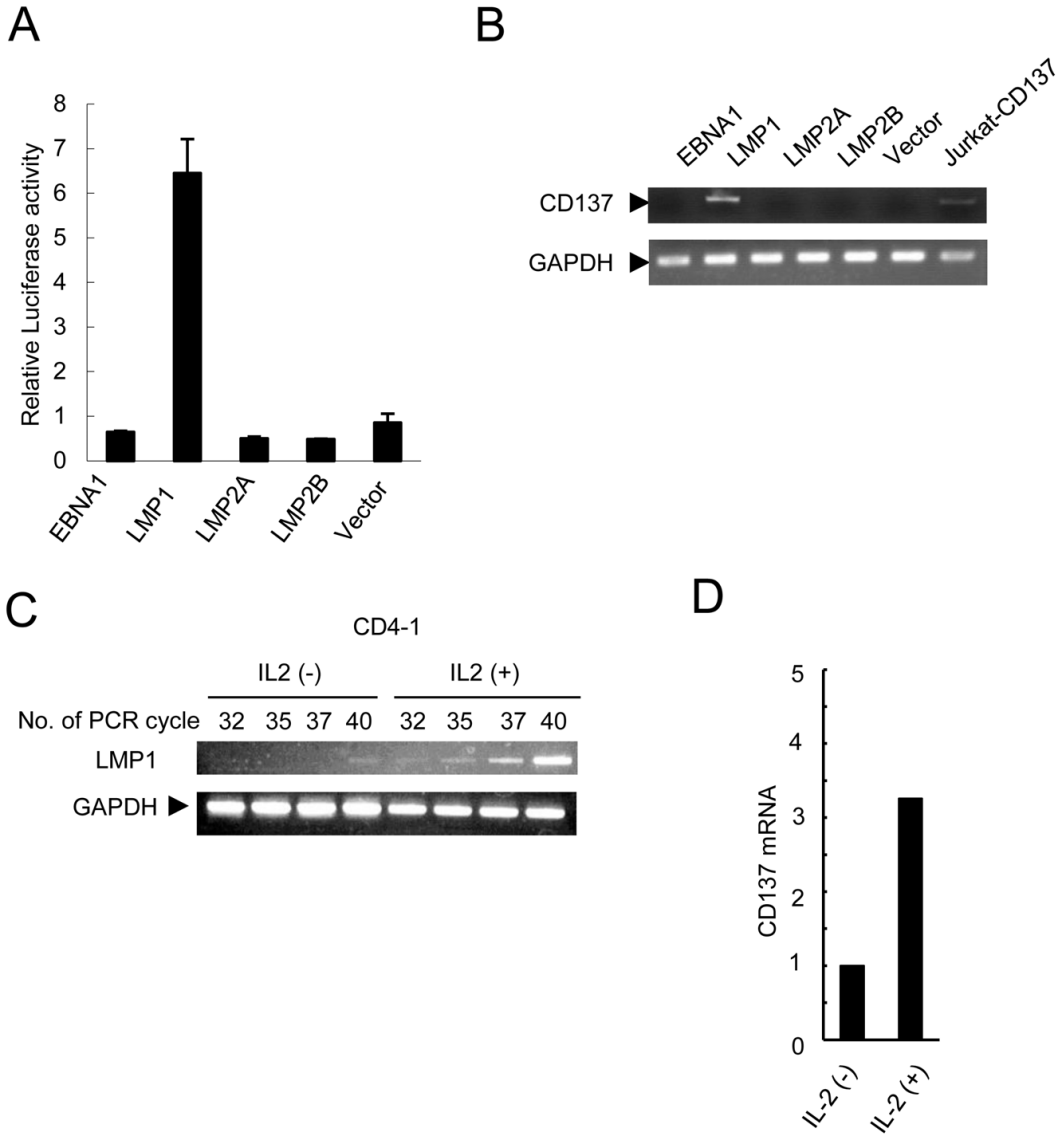
CD137 expression was upregulated by LMP1 whose expression was enhanced by IL-2 in EBV-T/NK-LPDs cells. (A) *CD137* transcription was examined using the assay described. Briefly, MOLT4 cells were transfected with 10 µg of the expression plasmids of the viral proteins, EBNA1, LMP1, LMP2A, LMP2B, or an empty vector as indicated, along with 10 µg of PGL3-4-1BB and 1 µg of pRLSV40. Twelve hours after transfection, the cells were harvested for a dual luciferase assay. Luciferase activity was normalized by *Renilla* luciferase activity and expressed in arbitrary units. The data are expressed as mean ± S.D. of 3 independent experiments. (B) MOLT4 cells were transfected with 10 µg of the expression plasmids of the viral proteins, EBNA1, LMP1, LMP2A, LMP2B, or an empty vector. Transcripts of CD137 (the upper panel) and GAPDH (the lower panel) in these cells were examined by RT-PCR. Jurkat-CD137 cells were used as a positive control. (C) RNAs were obtained from PBMCs from a EBV-T/NK-LPDs patient (CD4-1) which had been cultured with or without IL-2 for 3 days. Semi-quantitative RT-PCR assay for *LMP* was performed. Transcripts of *LMP1* (the upper panel) and *GAPDH* (the lower panel) were presented. (D) Transcripts of *CD137* and *GAPDH* were quantified by real time RT-PCR for the sample of 4C. Relative copy number was obtained by normalizing the *CD137* transcripts to those of *GAPDH*.

We investigated the mechanism of enhanced-CD137 expression by IL-2. First we performed luciferase reporter assay with a plasmid containing the *CD137* gene promoter. As shown in Figure 2A, EBV-infected MOLT4 cells were shown to express EBV-encoded proteins including LMP1, and EBNA1, considered to be latency type 2. So, MOLT4 cells were cotransfected with expression plasmids capable of expressing either of EBV-encoded proteins, LMP1, LMP2A, LMP2B or EBNA1. As shown in Figure 4A, LMP1 induced significant upregulation of *CD137* promoter activity, whereas the other molecules did not. Furthermore, in a transient expression assay with these viral proteins in MOLT4 cells, transcription of endogenous *CD137* mRNA was detected only in the LMP1-transfected cells (Figure 4B). These results indicated that, among the EBV proteins, LMP1 transactivated *CD137* expression in T and NK cells. Next we examined whether LMP1 expression was enhanced by IL-2 and might contribute to upregulation of CD137 expression in patients' cells. We isolated PBMCs from EBV-T/NK-LPDs patient (CD4-1) and cultured them with or without IL-2. As shown in Figure 4C, semi-quantitative RT-PCR demonstrated that *LMP1* mRNA was increased in IL-2-treated PBMCs. *CD137* mRNA was also increased in the IL-2-treated cells (Figure 4D). To confirm the *in vivo* contribution of IL-2 for CD137 expression, we examined the serum concentration of IL-2 in 7 EBV-T/NK-LPDs patients and 5 healthy donors. The concentration in the patients was 0.9-2.4 U/mL in 6 of 7 patients, whereas it was undetectable in 4 of 5 healthy donors (Table 2). These results suggested that CD137 expression was enhanced in the presence of IL-2 most likely through enhanced-expression of LMP1 in EBV-T/NK-LPDs patient cells.

**Table 2:** IL-2 concentration of the serum from EBV-T/NK-LPD patients.

EBV-T/NK-LPD (U/ml)		Healthy donor IL-2 (U/ml)
Case	IL-2 (U/ml)	
CD4-2	<0.8	<0.8
CD4-3	1.9	<0.8
CD4-5	0.9	<0.8
CD4-6	2.4	<0.8
CD8-2	2.1	1
CD8-3	1.1	
CD56-2	0.9	
CD56-3	0.9	

The concentration of IL-2 of the serum from EBV-T/NK-LPDs patients and from healthy donors. The lowest detection limit was 0.8 U/ml.

### CD137 was detected in EBV-positive cells infiltrating in the tissue lesion of EBV-T/NK-LPDs xenograft model

**Figure 5:**
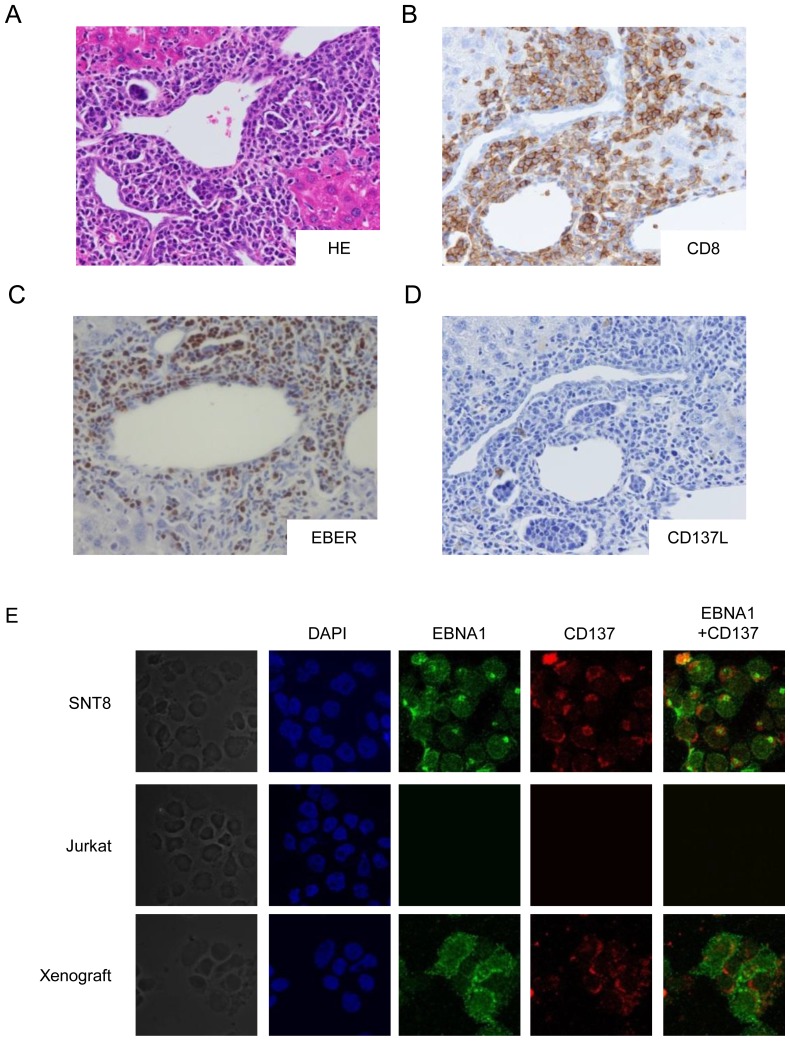
Histopathological specimen from the liver of the xenograft models. We generated the models by transplanting the cells from CD8-3 patient. Nine mice were examined and the representative data were shown. (A) Hematoxylin and eosin staining showed periportal infiltration of lymphocytes. (B) Immunochemical staining with anti-CD8 antibody (brown) showed that the infiltrating lymphocytes were positive for CD8. (C) *In situ* hybridization of Epstein–Barr virus-encoded mRNA (EBER) (brown). Infiltration of EBV-positive cells was detected in the periportal space. (D) Immunochemical staining with anti-CD137L antibody (brown) showed that CD137L-positive cells existed in the periportal space although the number of the cells was smaller than that of EBER positive cells. (original magnification, ×400). (E) Immune-fluorescent staining with anti-EBNA1 and anti-CD137 antibodies of cells isolated from the lesions. Mononuclear cells were obtained from the tissue lesions of a model mouse, stained with the antibodies. The cells were analyzed by confocal microscopy.

Next, we examined the CD137 expression on the EBV-positive cells infiltrating into the tissue of EBV-T/NK-LPDs. Since we could not perform the examination for human specimen due to difficulty of obtaining the samples, we used the xenograft models generated by intravenous injection of PBMCs from CD8-3 patient [15]. The injected cells were 2×10^6^ in number for each mouse and include CD8-positive EBV-infected cells with clonally proliferation from CD8-3 patient. EBV DNA load of the infected cells were more than 1.0×10^4^ copies/µgDNA. After engraftment, which was defined as detection of EBV DNA in the PB of the model, we performed autopsy. Nine mice were examined and the representative data were shown. As shown in Figure 5A–D, infiltration of EBV-positive and CD8-positive cells into the periportal regions in the liver was detected. 79.2% (396/500) of the infiltrating cells were EBER-positive, and 77.4% (387/500) of the cells were CD8-positive. These results indicated that most infiltrating cells were both positive for CD8 and EBER. Although CD137L-positive cells were also detected in the lesion, the number was markedly smaller than that of EBV-positive cells (Figure 5D). In order to determine CD137 expression on EBV-infected cells, we performed immune-fluorescent staining for the infiltrating cells in the lesions. As shown in Figure 5E, EBNA1-positive and CD137-positive cells were detected in the cells isolated from the lesions. LMP1 expression was confirmed in them (Figure S3). These results indicated that the infiltrating EBV-positive cells were both CD8- and CD137-positive.

### Stimulation of CD137 decreases etoposide–induced cell death of EBV-T/NK cells

**Figure 6:**
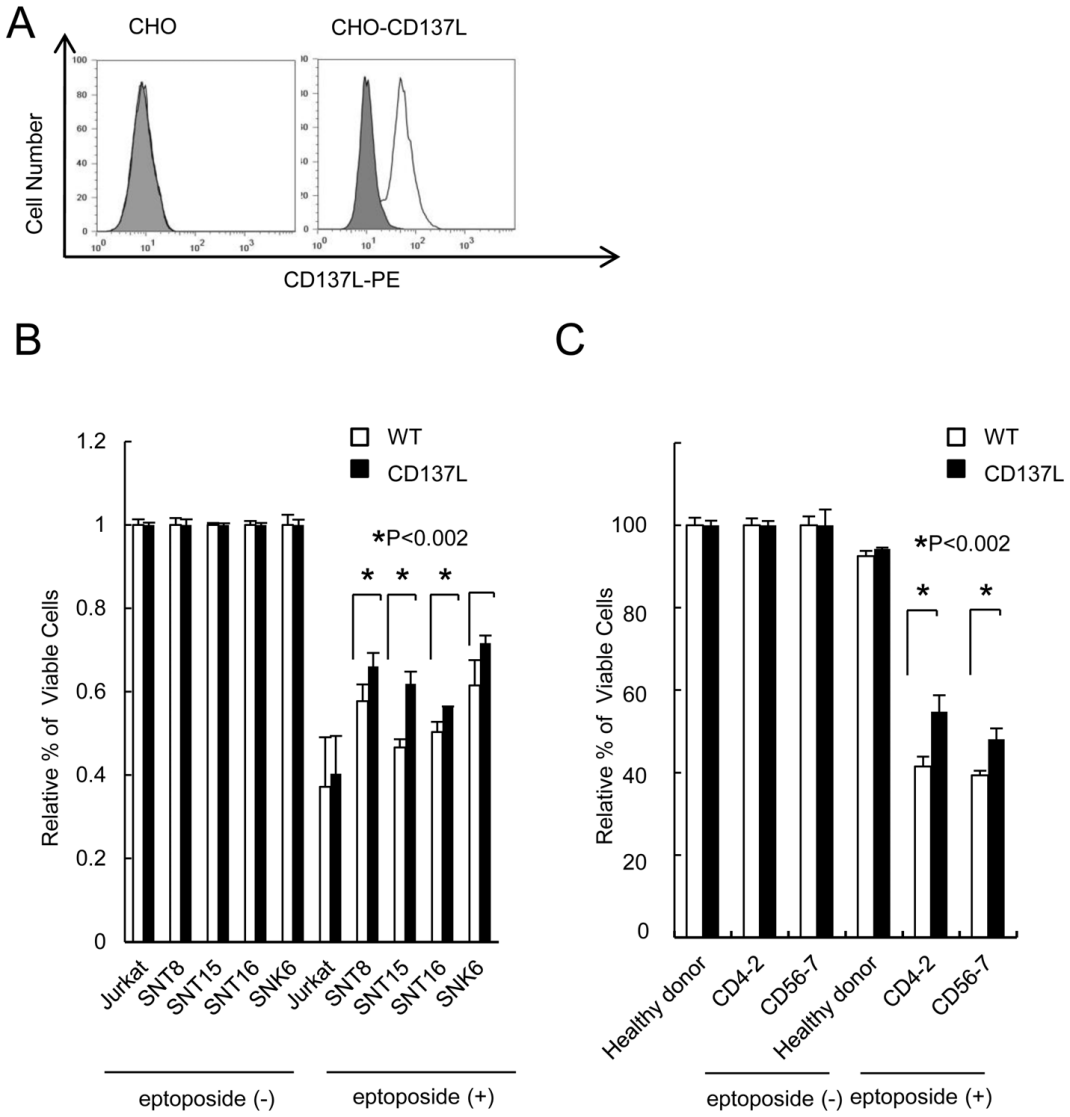
Stimulation of CD137 decreases etoposide-induced cell death of cells from patients with EBV-T/NK-LPDs. (A) CD137L expression on control Chinese Hamster Ovary (CHO) and CHO-CD137L cells. The expression was analyzed by flow cytometry using an antibody to CD137L (open histogram) or isotype-matched control immunoglobulin (gray, shaded histogram). (B) Jurkat cells and EBV- positive T- or NK-cell lines were cultured with 175 U/ml of IL-2 for 48 hours. Then they were cultured on control CHO or CHO-CD137L cells, which had been stained with PKH-26, with or without 2 µM of etoposide for 48 hours. They were then removed for assessment of viability. The cells were stained with DiOC6 and living EBV-T/NK-LPDs cells were detected as PKH-26-negative and DiOC6-positive cells by flow cytometry. The graph chart represents the relative numbers of living cells normalized by those of control cells which were cultured without etoposide. The data are expressed as mean ± S.D. of 3 independent experiments. (C) The PBMCs of EBV-T/NK-LPDs patients and healthy donors were cultured with 175 U/ml of IL-2 for 48 hours. Then they were cultured on control CHO or CHO-CD137L cells. They were then removed for assessment of viability as in B. The graph chart represents the relative numbers of living cells normalized by those of control cells which were cultured without etoposide. The data are expressed as mean ± S.D. of 3 independent experiments.

To explore the contribution of CD137 expression on EBV-T/NK cells to the development of EBV-T/NK-LPDs, we investigated the effects of CD137 on the survival. CHO-CD137L cells with stable expression of human CD137L on their surface were prepared for CD137 stimulation of EBV-T/NK cells (Figure 6A).

First we performed the assay for EBV-positive T- and NK-cell lines. We cocultured the cells with PKH-26-stained CHO cells in the presence of IL-2 with or without etoposide. Jurkat cells were used as a negative control. After the time indicated, we removed the cells and determined the number of living cells by detecting PKH-26 and DiOC6. PKH-26-negative cells were EBV-positive T/NK-cells and Jurkat cells. DiOC6-positive cells were living cells. In the presence of etoposide, the relative number of living EBV-positive T/NK-cells cultured with CHO-CD137L cells was significantly higher than that cultured with control CHO cells (Figure 6B). In contrast, T-cell line Jurkat cells, on which CD137 was not detected (Figure 1B), did not show a difference when cocultured with the 2 types of CHO cells (Figure 6B). In the absence of etoposide, CD137L had no significant effect on the viability of these cells (Figure 6B).

Next we performed the same assay for the primary cells from EBV-T/NK-LPDs patients. We cocultured PBMCs from 2 patients, CD4-2 and CD56-7 with PKH-26-stained CHO cells in the presence of IL-2 with or without etoposide. In the presence of etoposide, the relative number of living cells from EBV-T/NK-LPDs patients cultured with CHO-CD137L cells was significantly higher than that cultured with control CHO cells (Figure 6C). In contrast, cells form a healthy donor did not show a difference when cocultured with the 2 types of CHO cells (Figure 6C). These findings indicated that stimulation of CD137 significantly suppressed etoposide-induced cell death of the EBV-T/NK-LPDs cells.

## Discussion

CD137 is expressed following activation of T or NK cells and mediates molecular signals for proliferation, survival, and cytokine production by acting as a costimulatory molecule of the CD3-TCR complex [11], [16], [17]. However, few data for its roles in development of T or NK cell neoplasms have been reported to date. In this study we examined EBV-positive T or NK cells, and demonstrated that not only the cell lines but also freshly isolated cells of EBV-positive fractions from EBV-T/NK-LPDs patients expressed high levels of *CD137* mRNA. CD137 expression was also detected in EBV-positive cells isolated from the tissue lesions of EBV-T/NK-LPDs xenograft models. We demonstrated that EBV could directly induce CD137 expression most likely through LMP1 in T and NK cells. In addition, stimulation of CD137 by its ligand could suppress etoposide-induced cell death in EBV-positive and CD137-expressing T or NK cells. These results suggested that EBV could promote survival of T and NK cells by inducing CD137 and might be a cause for EBV-T/NK-neoplasms.

In the present study, *CD137* gene expression was significantly higher in freshly isolated EBV-positive T or NK cells from PB of patients compared with lymphocytes from healthy donors. *In vitro* IL-2 treatment enhanced CD137 expression in the EBV-infected cells of the patients, whereas not in control cells of the healthy donors. IL-2 treatment also increased *LMP1* gene expression in EBV-positive cells of EBV-T/NK-LPDs. Takahara and colleagues previously reported that IL-2 enhanced LMP1 expression in EBV-positive ENKL cell lines [18]. Since *CD137* promoter activity was enhanced by LMP1, we suggested that IL-2-induced CD137 protein expression was mediated by LMP1. In addition, the concentration of IL-2 in the serum of EBV-T/NK-LPDs patients was higher than that of healthy donors. Actually the concentration was lower than that of the culture medium, which we used in the assay. Ohga and colleagues, however, reported that the transcription of *IL-2* gene was upregulated in EBV-positive T- or NK-cells [19]. This finding suggested that the level might be high in the tissue lesion where large amount of EBV-positive T- or NK-cell were infiltrating. We detected CD137 protein expression in EBV-positive cells isolated from the lesion. The high expression level of *CD137* mRNA in the circulating EBV-positive cells may contribute to rapid and strong induction of the protein expression in the lesions.

We suggested that EBV enhanced *CD137* mRNA expression through LMP1. Expression level of LMP1 in ENKL is actually variable and other factors, such as miRNA, may play roles for lymphomagenesis in EBV-positive T- or NK-neoplasms [20]. However, all EBV-positive T- or NK-cell lines examined in the present study, expressed LMP1 according to our results (data not shown) and the report [14]. LMP1 activates c-JUN N-terminal kinase (JNK) [21], p38 mitogen-activated kinase (p38) [22], and Erk [23], which mediate the AP-1-activating pathway, and also activates NF-κB [24]. It was reported that CD137 expression was regulated by AP-1 and NF-κB in activated T cells [25]. LMP1 can, therefore, induce CD137 expression through AP-1 and NF-κB in T cells. In addition, we reported previously that EBV infection induced ectopic CD40 expression in T-cells [26], [27]. CD40 is known to activate NF-κB, JNK, p38 and Erk [28], [29]. Also, CD40-induced CD137 expression was recently reported [30]. These results indicate that EBV-induced CD137 expression can be mediated by LMP1, directly as well as through CD40.

Some questions, however, remain to be answered. The first concerns the localization of the CD137L. CD137L expression is induced in T cells when they are activated. [10] Its expression is also detected on various cancer cells [31]. Furthermore, expression of CD137 and CD137L is induced by the viral protein, Tax in ATLL cells and mediates autocrine survival signals, leading to proliferation of the infected cells and tumor development [12]. We therefore investigated CD137L expression on EBV-T/NK-cells themselves. However, we could not detect CD137L expression clearly on the surface of EBV-T/NK-LPDs cells. CD137L expression is usually detected not only on the surface of activated B and T cells, but also on antigen-presenting cells (APCs) such as dendritic cells, monocytes, and macrophages [32], [33]. EBV-negative cells, including histiocytes and macrophages are detected in EBV-T/NK-LPDs lesions surrounding EBV-infected cells [3]. These cells may express CD137L on their surface. Interestingly, CD137L-positive cells were certainly present in the lesions of EBV-T/NK-LPDs (Figure 5D). Since the number of CD137L-positive cells was markedly smaller than that of EBV-positive cells, they were considered to be different cell types. As we previously described, we generated the models by injection of the PBMCs from the patients [15]. Further investigations is required to determine the phenotype of the CD137L-positive cells in the lesions and to clarify whether these cells have some effects on EBV-positive cells, thereby contributing to disease progression. In addition, soluble CD137L (sCD137L) needs to be investigated. sCD137L is produced by lymphocytes or monocytes, with studies showing that it is present in PB of healthy donors and its level is increased in that of patients with hematological malignancies [34] and autoimmune diseases [35]. sCD137L may also have a role in hematopoietic neoplasm development, with its serum levels potentially being a prognostic factor in acute myeloid leukemia and myelodysplastic syndrome [36].

The next question is the actual role of CD137 in the disorders. EBV-T/NK neoplasms are not only lymphoid malignancies, but also have aspects of severe inflammatory diseases accompanied by high fever, cytokinemia, hemophagocytic syndrome and so on [3], [18], [37]-[39]. As CD137 mediates survival, proliferation, and cytokine production of CD137-expressing T cells, it may cause inflammation associated with the disease. In addition, CD137 acts as a “ligand” for CD137L. CD137L stimulation by CD137 also mediates intracellular signaling in CD137L-expressing cells [40]. In monocytes expressing CD137L, stimulation of the molecule induces proliferation and differentiation into DCs [41], [42]. In B cells expressing CD137L, the stimulation induces proliferation, differentiation and production of immunoglobulins [43], [44]. EBV-T/NK-neoplasms are associated with local and systemic inflammation, cytokinemia, or polyclonal gammopathy [38], [39]. CD137 may therefore contribute to disease development by inducing not only survival of the infected cells but also inflammation. Inhibition of CD137-mediating signals by targeting CD137 or CD137L should be conducted in order to clarify their roles.

It is well known that CD137 activates survival-promoting molecules including NF-kB in activated T cells [10]. However, the role of the CD137-CD137L interaction *in vivo* is still controversial. Recently, an agonistic CD137 antibody was created and used for xenograft models of human disease, cancer, or autoimmune diseases. In some mouse cancer models, agonistic CD137 antibody induces tumor suppression by upregulating the immune reaction of cytotoxic T-cells against tumor cells [45], [46]. On the other hand, in disease models of hyperimmune reactions such as asthma, GVHD, and autoimmune disease, the same antibody had the effect of suppressing T cells [47]. These findings show that CD137 regulates T-cell reactions both positively and negatively, and that the mechanism of the action *in vivo* is extremely complicated. As mentioned previously, EBV-T/NK-LPDs have two aspects: suppressed immune-reaction against EBV-T/NK-cells and a hyper-immune reaction as an inflammatory disease. The conflicting roles of the CD137–CD137L axis may be compatible with these clinical findings of EBV-T/NK-LPDs.

Our results indicate that upregulation of CD137 expression through LMP1 by EBV promotes cell survival in T or NK cells. This effect may contribute to the development of EBV-T/NK-neoplasms and suggests an attractive therapeutic target for the diseases.

## Materials and Methods

### Cells and reagents

The EBV-positive T/NK-cell (EBV-T/NK cell) lines SNT8, SNT15, SNT16, SNK1, SNK6, and SNK10 were cultured in RPMI containing 10% FCS and 175 U/ml of human IL-2 [14]. The EBV-negative T- and NK-cell lines, Jurkat, MOLT4, HPB-ALL, and MTA were cultured in RPMI containing 10% fetal calf serum (10% FCS-RPMI), whereas the EBV-negative NK-cell line, KHYG1 was cultured in 10% FCS-RPMI containing 175 U/ml of human interleukin-2 (IL-2). The B- cell lines, BJAB, Ramos, Raji, MD901 [48], HS-Sultan, and LCL were cultured in RPMI containing 10% FCS-RPMI. Jurkat, MOLT4, BJAB, Ramos, HS-Sultan and Raji cells were obtained from the American Type Culture Collection. LCL was established as previously described [26]. The expression of the viral proteins in LCL was demonstrated in Figure S4. MTA cells were obtained from Japanese Collection of Research Bioresources Cell Bank. Jurkat-CD137 and Chinese Hamster Ovary (CHO)-CD137L were generated as previously described [30]. Human recombinant IL-2 was purchased from R&D systems (Abington, UK) and etoposide from Wako (Osaka, Japan).

### PCR assay for CD137

The sequences of the PCR primers used for detection of the CD137 gene were as follows: forward, 5′-GTGCCAGATTTCATCATGGG-3′ (exon 2 of CD137) and reverse, 5′-CAACAGCCCTATTGACTTCC-3′ (exon 9 of CD137). The expression levels of the CD137 gene were determined by quantitative PCR, as described previously [13].

### Diagnosis of EBV-T/NK-LPDs

EBV-T/NK-LPDs was diagnosed according to the following criteria: the presence of characteristic symptoms, an increase in EBV DNA load in peripheral blood (PB), and the detection of clonally proliferating EBV-positive T or NK cells [4], [49].

### Detection and isolation of EBV-positive cells in EBV-T/NK-LPDs patients

Detection and isolation of EBV-infected cells were performed as described previously [27]. Briefly, peripheral blood mononuclear cells (PBMCs) from EBV-T/NK-LPDs patients were isolated by density gradient centrifugation using Separate-L (Muto Pure Chemical, Tokyo, Japan) and sorted into CD19-, CD4-, CD8-, or CD56-positive fractions by antibody-conjugated magnetic beads (IMag Human CD19, 4, 8, and 56 Particles-DM; BD Biosciences, Sparks, MD, USA). The fraction which was negative for these markers was considered γδ T cell fraction. The EBV DNA load in each fraction was then measured by the real-time RT-PCR [50] on the basis of the TaqMan system (Applied Biosystems, Foster City, CA, USA). The fraction with the highest titer was assumed to be that with EBV-positive cells. In order to examine *CD137* mRNA expression in the infected cell, we isolated EBV-positive cells from PBMCs by magnetic beads conjugating antibodies for the surface markers of the infected cells.

### Antibodies

Mouse antihuman CD137-PE, CD4-FITC, CD8-FITC, CD56-FITC and CD137L-PE as well as their control isotype antibodies were purchased from Becton, Dickinson and Company (Franklin Lakes, NJ, USA).

### In vitro EBV infection assay

MOLT4 cells were infected with EBV as described previously [26]. Briefly, EBV was prepared from culture medium of B95-8 cells as described [51], and then concentrated (200-fold) in RPMI medium 1640 supplemented with 10% FCS. The virus suspension was filtered (0.45 µm) and the recipient cells (2×10^6^ to 1×10^7^) were incubated in 1 or 5 ml of the suspension for 1 h, and then rinsed twice with culture medium (10% RPMI). The efficiency of infection was >90% as judged by EBNA1 staining. For inactivation of the EBV genome, 1 ml of virus suspension in a 100-mm dish was irradiated with UV (254 nm) at 1 Jµcm^2^ using a FUNA-UV-LINKER FS-800 (Funakoshi, Tokyo). Infection was verified by EBV DNA quantification, and immune fluorescence staining of EBNA1 staining of the cells as described using Polyclonal Rabbit Anti-Human C3c Complement/FITC antibody (Dako, Glostrup, Denmark) [52].

### PCR assay for EBV proteins

RT-PCR for detection of mRNA for the viral proteins, *LMP1, LMP2A, LMP2B* and *EBNA1* was performed according to the previous report [15].

### Plasmids

The reporter plasmid PGL3-4-1BB for the detection of *CD137* promoter activation was kindly provided by Dr. Pichler [12]. The reporter plasmid for detection of NF-κB activation, pNF-κB-Luc, was purchased from Stratagene (Santa Clara, CA, USA), and the control *Renilla* luciferase plasmid pRL-SV40 from Promega (Madison, WI, USA). Plasmids containing EBV-encoded proteins, LMP1, LMP2A, LMP2B and EBNA1 were generated from the EBV-infected cell line B95-8 [53].

### Luciferase reporter assays

The assays of transiently transfected cells were performed as described previously [54].

### Measurement of serum IL-2

The concentration of IL-2 in the serum was examined by SRL, Inc. (Tokyo, Japan) using enzyme-linked immunosorbent assay (ELISA). The lowest detection limit was 0.8 U/ml.

### Generation of the xenograft model of EBV-T/NK-LPDs

Male NOD/Shi-scid/IL-2Rγnull (NOG) mice were obtained from the Central Institute for Experimental Animals (Kawasaki, Japan) and maintained under specific pathogen-free conditions. The model was generated by injection of PBMCs from patients to six weeks old mice through the tail vein as described previously [15]. Intravenous anesthesia by tribromoethanol was performed in order to minimize suffering. Engraftment was determined by detecting EBV DNA in the peripheral blood. After engraftment, mice were euthanized via CO_2_ inhalation and applied for pathological and virological analyses.

### Immunohistochemistry

The 4 µm thick paraffin-embedded formalin-fixed tissue sections were de-paraffinized, and heat-based antigen retrieval was performed in 0.1 M citrate buffer (pH 6.0). Endogeneous peroxidase activity was inhibited using hydrogen peroxide. The primary antibodies for CD137 (ab3169) and CD137L (ab64912) were purchased from Abcam (Cambridge, MA, USA). The detection system was the streptavidin-biotin-peroxidase complex technique (ABC kit; Vector Laboratories, Burlingame, CA, USA) with diaminobenzidine (DAB; Nichirei Bioscience, Tokyo, Japan) as the chromogen. *In situ* hybridization (ISH) of Epstein–Barr virus-encoded mRNA (EBER) was performed for detection of EBV in tissue sections by Epstein-Barr Virus (EBER) PNA Probe/Fluorescein (DAKO, Carpinteria, CA, USA) and second antibody for Fluorescein (Dako, Glostrup, Denmark).

### Immune-fluorescent staining

The expression of CD137 protein on EBV-infected cells was examined by immune-fluorescent staining. Cells were fixed on slides by immersing in 4% paraformaldehyde for 10 min, followed by washing three times in PBS and incubation with mouse monoclonal anti-CD137, goat polyclonal anti-EBNA1) antibodies (Abcam, Cambridge, MA, USA), Cy5-conjugated Affinipure donkey anti-mouse antibody, and FITC-conjugated donkey anti-goat antibody (Jackson ImmunoResearch Laboratories, Inc. PA, USA). Nuclei were counterstained with ProLong Gold and DAPI (Invitrogen, Carlsbad, CA, USA), and the cells were analyzed by confocal microscopy (Fluoview FV10i, Olympus).

### Stimulation of CD137 by ligand-expressing cells and detection of cell viability

The PBMCs were isolated from patients of EBV-T/NK-LPDs. Control CHO or CHO-CD137L cells were stained with PKH-26 (PKH-26 Red Fluorescent Cell Linker Kit; Sigma-Aldrich, St. Louis, MO, USA) according to the manufacturer's instructions, and plated on the wells. The PBMCs were then overlaid on pre-seeded control CHO or CHO-CD137L cells, and cultured with or without etoposide in 10% FCS-RPMI containing 175 U/ml of IL-2. After 48 h incubation, the cells were stained with DiOC6 (Invitrogen, Carlsbad, CA, USA) and removed. The cells were analyzed using a FACS Calibur flow cytometer (Becton, Dickinson and Company, Franklin Lakes, NJ USA), with PKH-26–negative and DiOC6-positive cells considered as living EBV-T/NK cells.

### Statistical analysis

For statistical analyses of Figure 3A and 3B, Mann-Whitney test was performed using GraphPad Prism 5 (GraphPad Software, La Jolla, CA, USA). Student *t* test was performed for Figure 6B and 6C.

The study complied with the principles of the Declaration of Helsinki and was approved by the ethical committee of Tokyo Medical and Dental University (TMDU). Written informed consent was obtained from each patient. The experiments with NOG mice are in accordance with the Guidelines for Animal Experimentation of the Japanese Association for Laboratory Animal Science, as well as ARRIVE guidelines [55]. The experiments were approved by the Institutional Animal Care and Use Committee of TMDU (No. 0140087A).

## Supporting Information

Figure S1 **CD137L expression in EBV-positive cell lines.** Surface expression of CD137L was examined by flow cytometry using an antibody to CD137L (open histogram) or isotype-matched control immunoglobulin (gray, shaded histogram). The mean fluorescent intensity of CD137 was normalized by that of isotype-matched control and expressed as MFIR (mean fluorescence intensity rate) in arbitrary units. CHO-CD137L cells were used as positive control.
(TIF)

Figure S2 **CD137 expression in PBMCs from EBV-positive T-NK-lymphoproliferative patients and those from healthy donors (HD).** After collection, the cells were cultured with IL-2 for 3 days. The expression was analyzed by flow cytometry using an antibody to CD137 and to surface protein expressed on EBV-positive cells.
(TIF)

Figure S3 **Immune-fluorescent staining with anti-LMP1 antibody of cells isolated from the lesions.** Mononuclear cells were obtained from the tissue lesions of a model mouse, stained with the antibody. The cells were analyzed by confocal microscopy.
(TIF)

Figure S4 **LCL that we used in the study was established as previously described** [26] **.** The infection was confirmed by RT-PCR for EBNA. We also examined and detected the expression of the lytic protein, BZLF1 [56]. Akata cells [57] stimulated with IgG were used as a positive control for BZLF1 expression. Since BZLF1 was not expressed in them, we concluded that the infection was latent.
(TIF)
