# Supplementary figures and images for: CD137 Expression Is Induced by Epstein-Barr Virus Infection through LMP1 in T or NK Cells and Mediates Survival Promoting Signals
Source: PLoS One. 2014 Nov 19;9(11):e112564. doi: 10.1371/journal.pone.0112564 (PMC4237363; doi:10.1371/journal.pone.0112564)

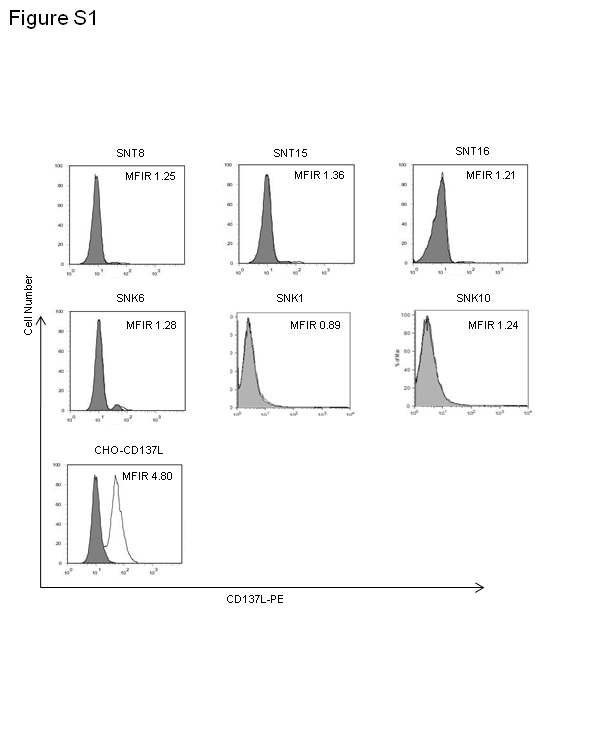

Supplement: Figure S1 — CD137L expression in EBV-positive cell lines. Surface expression of CD137L was examined by flow cytometry using an antibody to CD137L (open histogram) or isotype-matched control immunoglobulin (gray, shaded histogram). The mean fluorescent intensity of CD137 was normalized by that of isotype-matched control and expressed as MFIR (mean fluorescence intensity rate) in arbitrary units. CHO-CD137L cells were used as positive control. (TIF) [file pone.0112564.s001.tif]

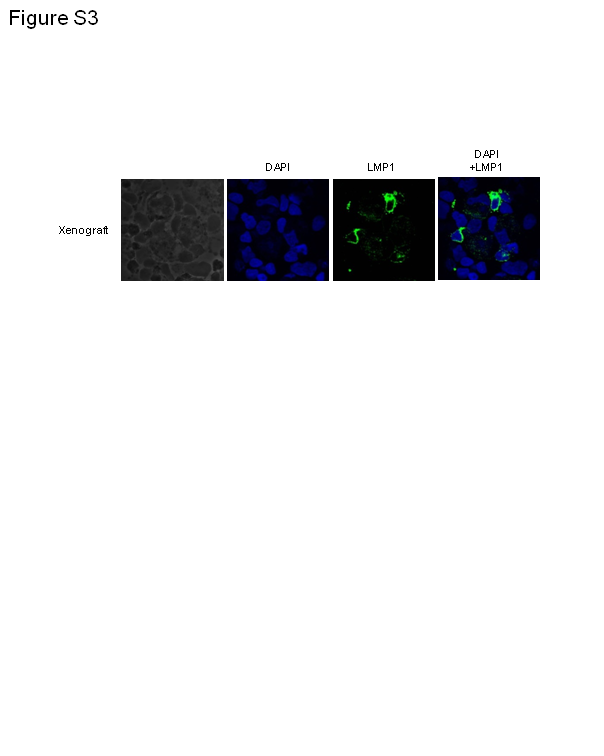

Supplement: Figure S3 — Immune-fluorescent staining with anti-LMP1 antibody of cells isolated from the lesions. Mononuclear cells were obtained from the tissue lesions of a model mouse, stained with the antibody. The cells were analyzed by confocal microscopy. (TIF) [file pone.0112564.s003.tif]
